# Cellulase activity and age-based variation of intestinal microbiota in Hezuo pigs

**DOI:** 10.3389/fmicb.2025.1599847

**Published:** 2025-05-09

**Authors:** Yuhao Liang, Fei Wang, Rui Jia, Jie Li, Longlong Wang, Yajuan Li, Yao Li, Shuangbao Gun, Qiaoli Yang

**Affiliations:** ^1^College of Animal Science and Technology, Gansu Agricultural University, Lanzhou, China; ^2^Gansu Research Center for Swine Production Engineering and Technology, Lanzhou, China; ^3^Gansu Diebu Juema Pig Science and Technology Backyard, Diebu, China

**Keywords:** pig, intestinal microbiota, fiber degradation, physiological maturity stage age, 16S rRNA sequencing

## Abstract

**Introduction:**

To examine the fiber-degrading enzyme activity and the changes of intestinal microbiota in Hezuo pigs across different age stages.

**Methods:**

Fecal samples from 36 semi-grazed Hezuo pigs across five growth stages were collected for cellulase activity and 16S rRNA gene sequencing analysis.

**Results:**

The results showed that the cellobiase activity in adult Hezuo pigs was markedly higher than in other groups (*p* < 0.01). The intestinal microbiota of Hezuo pigs was predominantly composed of Firmicutes and Bacteroidetes. The prevalence of Proteobacteria of nursing piglets was significantly higher compared to other stages (*p* < 0.05), which might be related to protein degradation. *Prevotella_NK3B31_group* was a shared dominant genus across at all age stages, while *Ruminococcaceae_UCG-005* dominated during the weaning, growth, fattening and adult stages. Its cellulose-degrading enzyme secretion capacity enhanced with age to meet the high-fiber dietary demands of Hezuo pigs. Functional prediction of intestinal microbiota in adjacent age groups using PICRUSt revealed that the differences between lactating piglets and weaned piglets were primarily due to the enrichment of various metabolic pathways functional genes, while differences between fattening pigs and adult sows were negligible. Adult boars showed significant enrichment in amino acid metabolism, energy metabolism, and nucleotide metabolism pathways compared to fattening piglets.

**Discussion:**

These results reveal the age-related dynamic development of intestinal microbiota in Hezuo pigs, providing novel insights into the mechanism of their roughage tolerance.

## 1 Introduction

The gastrointestinal tract of animals harbors a diverse and complex array of microflora, which plays a crucial role in maintaining the bodily health (Chen et al., 2021; Brown et al., 2023). They participate in the host’s metabolism, growth, development, and immunological control, and are intricately linked to the onset of numerous diseases (Ding and Xiao, 2020; Yu et al., 2024). The primary determinants influencing the microbial community in the gastrointestinal system of animals encompass genetics, nutrition, and lifestyle. Numerous studies have demonstrated substantial changes in the structure and function of the gut microbiota across different animal age stages. Li et al. Discovered that juvenile Hainan gibbons possess a greater proportion of bacteria from the Bacteroidetes in their intestinal tract, which enhances immune system function; conversely, adult Hainan gibbons predominantly harbor bacteria from the bacterial phylum of Firmicutes, facilitating superior digestion and nutrient absorption (Li et al., 2022). Yan et al. compared the intestinal flora across various age groups in the Chinese population, revealing that the intestinal tracts of the older individuals are enriched with bacteria such as *Bacteroides fragilis*, *Clostridium bolteae and Escherichia coli*, alongside other intestinal microorganisms that facilitate inflammation-induced aging. In contrast, the intestinal flora of the younger individuals exhibited a predominance of *Barnesiella intestinihominis* and *Parabacteroides merdae*, which may retard aging by producing short-chain fatty acids (Yan et al., 2022).

The diversity and composition of the intestinal microbial population in animals varied dramatically across different ages. The analysis of rumen microbiota structure and diversity in Mongolian cattle at three ages revealed that 5-month-old cattle had low abundance and a simplistic microbiota, mainly consisting *Anabacterium* and *Bifidobacterium*. In contrast, the rumen microbiota of 18- and 36-month-old cattle exhibited high abundance and complexity, dominated by*Rikenellaceae_RC9_gut_group* and *Alloprevotella*, which are recognized for short-chain fatty acid production and fiber degradation, respectively (Ahmad et al., 2022). Elevated levels of *Fusobacterium* and *Sutterella* in neonatal piglet intestines significantly enhance *Clostridium difficile* infection risk, with the likelihood of such infection diminishing with age as *Prevotella* spp. abundance increase, while *Fusobacterium* and *Sutterella* abundance declines (Proctor et al., 2021). A study on Szechuan black pigs revealed that adults exhibited a higher abundance of *Streptococcus* spp. in their digestive tracts than juveniles, which enhances intestinal immunity and helps prevent inflammation (He et al., 2023). These studies are crucial for comprehending the physiological alterations during livestock growth and development, optimizing feeding management strategies, preventing intestinal diseases, and developing probiotics to regulate intestinal microbial balance, and enhance livestock health and productivity.

The Hezuo pig, also known as the fern pig, is a native breed endemic to alpine pastoral regions, primarily production in the Gannan Tibetan Autonomous Prefecture, Gansu Province (Tang et al., 2023). This region, at an altitude of approximately 3,000 meters. Attributing to its cold plateau climate, diverse geomorphology, and geographic isolation, Hezuo pig has developed exceptional germplasm characteristics, including strong disease resistance, roughage tolerance, and cold tolerance (Zhang et al., 2022; Yin et al., 2024). Hezuo pigs primarily raised for pasture, grazing on the roots and leaves of fern, wild clover, dandelion, and other forage grasses, which contain higher fiber content compared to the corn-soybean meal rations of introduced pig breeds (Wang et al., 2024). Studies have shown that, in comparison to foreign breeds, local Chinese pig breeds exhibit superior ability of crude fiber digestion and utilisation, attributed to their enhanced intestinal fiber digestive enzymes, bacteria, and short-chain fatty acid metabolites (Pu et al., 2020; Niu et al., 2022). At a crude fiber level of 8%, the crude fiber digestibility of local Chinese pig breeds exceeded that of foreign introduced pig breeds by 27%. Previous studies conducted by the organisation indicated that the crude fiber digestibility of Hezuo pigs was about 31%. Animals lack endogenous cellulase in their digestive tracts, rendering them incapable of directly using cellulose. The symbiotic microbial population in the gastrointestinal tract of animals secretes cellulase, facilitating the decomposition of cellulose to supply energy for the animals (Marynowska et al., 2020). Nevertheless, limited research has been undertaken regarding the microbial community in the gut of Hezuo pigs, particularly the variation in gut microflora across different age stages. The function of intestinal bacteria in the digestion and utilisation of dietary fiber in Hezuo pigs requires further investigation.

This study was performed to investigate the changes of intestinal microbiota and the cellulase activity in Hezuo pigs across different age stages. The results aim to establish a reference for the comprehension of the Intestinal microbiota of Hezuo pigs at different growth and development stage sand their physiological roles, while also investigating their roughage tolerance.

## 2 Materials and methods

### 2.1 Experimental animals and sample collection

Hezuo pigs were maintained under semi-grazing management conditions by local farmers in Lexiu town, Hezuo City, Gannan Tibetan Autonomous Prefecture, Gansu Province, China. Lexiu Town is located in the northeastern edge of the Qinghai-Tibet Plateau. The area is dominated by alpine meadows with a coverage of more than 90%. The main vegetation is dominated by Gramineae and Cyperaceae plants. The common species are Elymus nutans Griseb., Stipa aliena Keng, Argentina anserine (L.) Rydb., *Cyperus rotundus* L., Medicago falcata L., and so on. A total of 36 individuals were selected across five developmental stages. The adult stage includes six adult sows (365–400 days old), six adult boars (365–400 days old), and six each of the remaining four growth stages: lactating piglets (21 days old), weaned piglets (60 days old), growing pigs (120–150 days old), and fattening pigs (180–240 days old). All sampling procedures were conducted in August 2023.

The experimental cohort was permitted to forage daily from 08:00 to 20:00 h on native vegetation, including the roots, leaves, and seeds of wild plants like bracken grass, wild clover, sedge, and plantains. From 8:00 a.m. to 20:00 p.m., the test pigs were allowed to graze Supplemental feed comprises 0.26 kg•head^−1^•d^−1^ grass meal, 0.05 kg•head^−1^•d^−1^ corn meal, and 0.18 kg•head^−1^•d^−1^ barley. The animal study was approved by the Animal Care and Use Ethics Committee of Gansu Agricultural University (approval number 2017-098). All methods are carried out in accordance with the international standards published in the Guidelines for the Feeding, Management, and Use of Laboratory Animals’ (8th edition) and the relevant guidelines and regulations.

Fecal sampling was conducted during postprandial defecation events occurring 2–5 min following feeding. Sterile collection protocols were implemented using DNA-free cotton swabs to obtain luminal fecal matter from the non-contact surface of freshly voided feces. Samples were immediately partitioned into 5 mL sterile polypropylene centrifuge tubes, promptly frozen in liquid nitrogen, and subsequently transferred to −80°C ultra-low temperature freezers for long-term cryopreservation until downstream analysis. Detailed sample metadata are presented in Table 1.

**Table 1:** Sample information and number.

Growth phase	Age (d)	Group	Number of samples (parts)
Suckling piglet	21	SP	6
Weaned piglet	60	WP	6
Growing pig	120 ~ 150	GP	6
Fattening pig	180 ~ 240	FP	6
Adult sow	365 ~ 400	AS	6
Adult boar	365 ~ 400	AB	6

### 2.2 Fiber degrading enzyme activity analysis

Fecal samples (0.5 g wet weight) were thawed and homogenized in a 0.9% (w/v) sodium chloride solution (NaCl, 9:1 v/w ratio) using ice-cold water immersion for 2 min. The homogenate was centrifuged at 3500 × g for 10 min at 4°C, and the aliquoted and stored at −20°C for subsequent enzymatic assays.

Enzyme activities of xylanase, cellobiase, carboxymethyl cellulase, and microcrystalline cellulase were quantified using commercial ELISA kits (Jiangsu Kotter Bio, Jiangsu, China; KT811231-A, KT811243-A, KT811235-A, KT811239-A) according to the manufacturer’s protocol. Briefly, reagents, standards, and supernatant were prepared under controlled conditions. Absorbance measurements were performed in triplicate at 450 nm using a microplate reader (Thermofisher, China), with activity calculations based on standard curves generated from p-nitrophenol (pNP) derivatives.

### 2.3 Sequencing analysis of fecal flora

#### 2.3.1 Extraction and detection of total DNA of fecal bacteria

Using the soil and fecal genomic DNA extraction kit for magnetic bead method (Beijing Tiangen Bio, Beijing, China, DP712-01), the samples’ whole genomic DNA was extracted. The DNA concentration and purity were then assessed on a 1% agarose gel and DNA concentration and purity were measured by spectrophotometry (NanoDrop 2000, Thermo Fisher Scientific). Based on the concentration, the DNA was diluted with sterile enzyme-free water to 1 ng/μL.

#### 2.3.2 16SrRNA high-throughput sequencing

The V3-V4 hypervariable region of the 16SrRNA gene was amplified via polymerase chain reaction (PCR) using bacterial genomic DNA as the template. Primers flanked by unique barcode sequences were employed, with the following oligonucleotide sequences: F: 5’-CCTAYGGGRBGCASCAG-3′, R: 5’-GGACTACNNGGGGTATCTAAT-3′. PCR reactions were performed in a 30 μL reaction volume containing: 15 μL of Phusion^®^ High-Fidelity PCR Master Mix (New England Biolabs, Ipswich, MA, United States, M0531S), 0.2 μM primers, and 10 ng of genomic DNA template. The initial denaturation was conducted at 98°C for 1 min, followed by denaturation at 98°C for 10 s, annealing at 50°C for 30 s, and 72°C for 30 cycles. The size of PCR amplification of all samples ranged from 405 to 426 nt.

PCR products were identified through electrophoresis on a 2% agarose gel stained with ethidium bromide. Amplicons of PCR products were purified using magnetic bead-based purification and quantified spectrophotometrically (NanoDrop 2000, Thermo Fisher Scientific). Equimolar pooling of PCR products was performed based on concentration measurements. The pooled library was subjected to a second round of electrophoresis on a 2% agarose gel, and the target bands were excised and purified using the universal DNA purification and recovery kit (Beijing Tiangen Bio, Beijing, China, DP214).

The NEBNext^®^ Ultra™ II FS DNA PCR-free Library Prep Kit (New England Biolabs, Ipswich, MA, United States, E7430S) was employed for library construction, which was then quantified using Qubit and qPCR qualification. NovaSeq6000 was employed for PE250 on-line sequencing performed by Beijing Novogene Biotechnology Co.

#### 2.3.3 Bioinformatics analysis

The data was segregated based on barcode sequence and PCR primer sequence. After truncating these sequences, reads were concatenated utilizing FLASH (Version 1.2.11, http://ccb.jhu.edu/software/FLASH/) (Abellan-Schneyder et al., 2021), resulting in raw tag data (raw tags). The spliced raw tags were meticulously processed with Fastp software (Version 0.23.1) to generate high-quality tag data (Clean Tags) (Nayman et al., 2023). Tag sequences were then checked with the species annotation database (Silva database https://www.arb-silva.de/ https://unite.ut.ee/ for 16S/18S, Unite database for ITS) to identify and eliminate chimeric sequences and finally obtain the effective data (Effective Tags) (Welzel et al., 2020). In the sequencing, the percentages of bases with base mass values greater than 20 (sequencing error rate less than 1%) and 30 (sequencing error rate less than 0.1%) in effective tags were more than 98 and 94%, respectively. The sequencing coverage of all samples was more than 99.5%.

PCoA analysis and sample dilution curve generation were performed using R software (version 4.0.3). Alpha diversity analysis (Chao1, Shannon, Simpson, and ACE indices) were conducted utilizing Qiime software (Version 1.9.1). Histograms depicting the distribution of the top 10 species by relative abundance at family, genus, and species taxonomic levels were generated for each sample group using the SVG function. Microbiota marker analyses were conducted using LEfSe software with a default LDA score cutoff of 4. The functional gene composition of the fecal microbiota was estimated using PICRUSt software. The parameters used in the bioinformatics analysis of each software program are shown in tabular form in Supplementary material 1.

#### 2.3.4 Statistical analysis of data

We employed SPSS 27.0 (Chicago, United States, version 27.0) for a one-way ANOVA on fiber digestive enzyme activity, presenting the results as mean ± standard deviation (mean ± SD). Significant differences between species of adjacent ages at the genus level were analyzed using the *t*-test in R software. Microbiota diversity was evaluated using the Kruskal-Wallis rank-sum test, and functional variations in the gut flora of Hezuo pigs of similar ages were assessed by the *t*-test.

## 3 Results

### 3.1 Analysis of cellulase activity of Hezuo pigs at different age stages

Table 2 compares cellulase activities in the feces of Hezuo pigs at various ages, revealing no significant differences in intestinal xylanase, carboxymethyl cellulase, and microcrystalline cellulase among weaned, growing, fattening, and adult pigs (*p* > 0.05). However, cellobiose activity differed significantly, with adult gilts exhibiting higher levels than the other groups (*p* < 0.01), weaned piglets had significantly lower intestinal fibrous disaccharide activity compared to fattening pigs and adult gilts (*p* < 0.01), In addition, highly significant differences were shown between growing and fattening pigs (*p* < 0.01).

**Table 2:** Analysis of fibrous digestive enzyme activities of Hezuo pigs at different developmental stages.

Fibrous digestive enzyme	WP	GP	FP	AS	AB	*p* value
Xylanase (U/L)	25.48 ± 5.29	26.59 ± 5.81	25.58 ± 5.04	22.28 ± 10.60	25.48 ± 5.293	0.71
Cellobiase (U/L)	32.70 ± 9.43^D^	38.52 ± 6.48^CD^	45.55 ± 7.70^C^	55.33 ± 12.44^B^	67.80 ± 6.20^A^	0.001
CMCase (U/L)	30.23 ± 5.95	30.29 ± 6.51	40.44 ± 5.93	34.46 ± 4.31	42.67 ± 3.45	0.51
MCCase (U/L)	11.35 ± 3.14	11.59 ± 3.49	12.90 ± 2.81	14.38 ± 4.05	12.02 ± 3.29	0.177

Peer data labeled with the same letter or no letter on the shoulder indicate non-significant differences (*p* > 0.05), while different capital letters indicate highly significant differences (*p* < 0.01).

### 3.2 Sequencing data and diversity analysis of intestinal microbiota in Hezuo pigs at different age stages

#### 3.2.1 Quality control data of 16S rRNA gene sequencing

Using the Illumina NovaSeq sequencing platform, we obtained 4,778,881 raw PE sequences from 46 fecal samples. These sequences were then processed to generate 3,498,909 high-quality tags after splicing, quality control, and chimera filtering. The mean number of reads per sample was 76,063, with an average read length of 415 base pairs. As sequencing effort increased, observed species richness demonstrated a characteristic pattern of gradual increase followed by stabilization. This asymptotic trend suggests that the sequencing depth was to capture the majority of bacterial taxa present in the fecal microbiota of Hezuo pigs All raw sequencing data have been deposited in the NCBI Sequence Read Archive (SRA) database under BioProject PRJNA1199767 (accession URL: https://trace.ncbi.nlm.nih.gov/).

#### 3.2.2 OTU analysis and species annotation

All quality-filtered sequences were clustered into operational taxonomic units (OTUs) at a 97% similarity threshold using the UPARSE pipeline, enabling microbial community composition analysis. Taxonomic annotation was performed against the SILVA 138.1 reference database (release 138.1) with an 80% minimum bootstrap confidence threshold. 2, 920 distinct OTUs were identified in the fecal microbiota of Hezuo pigs across six developmental stages. Core microbiota analysis revealed 177 conserved OTUs present in all groups, while group-specific signatures comprised 111, 131, 171, 72, 123, and 135 unique OTUs in the groups SP (Suckling Piglet), WP (Weaned Piglet), GP (Growing Pig), FP (Fattening Pig), AS (Adult Sow) and AB (Adult Boar), respectively.

#### 3.2.3 Analysis of *α* diversity of fecal microbiota in Hezuo pigs across different ages

**Figure 1:**
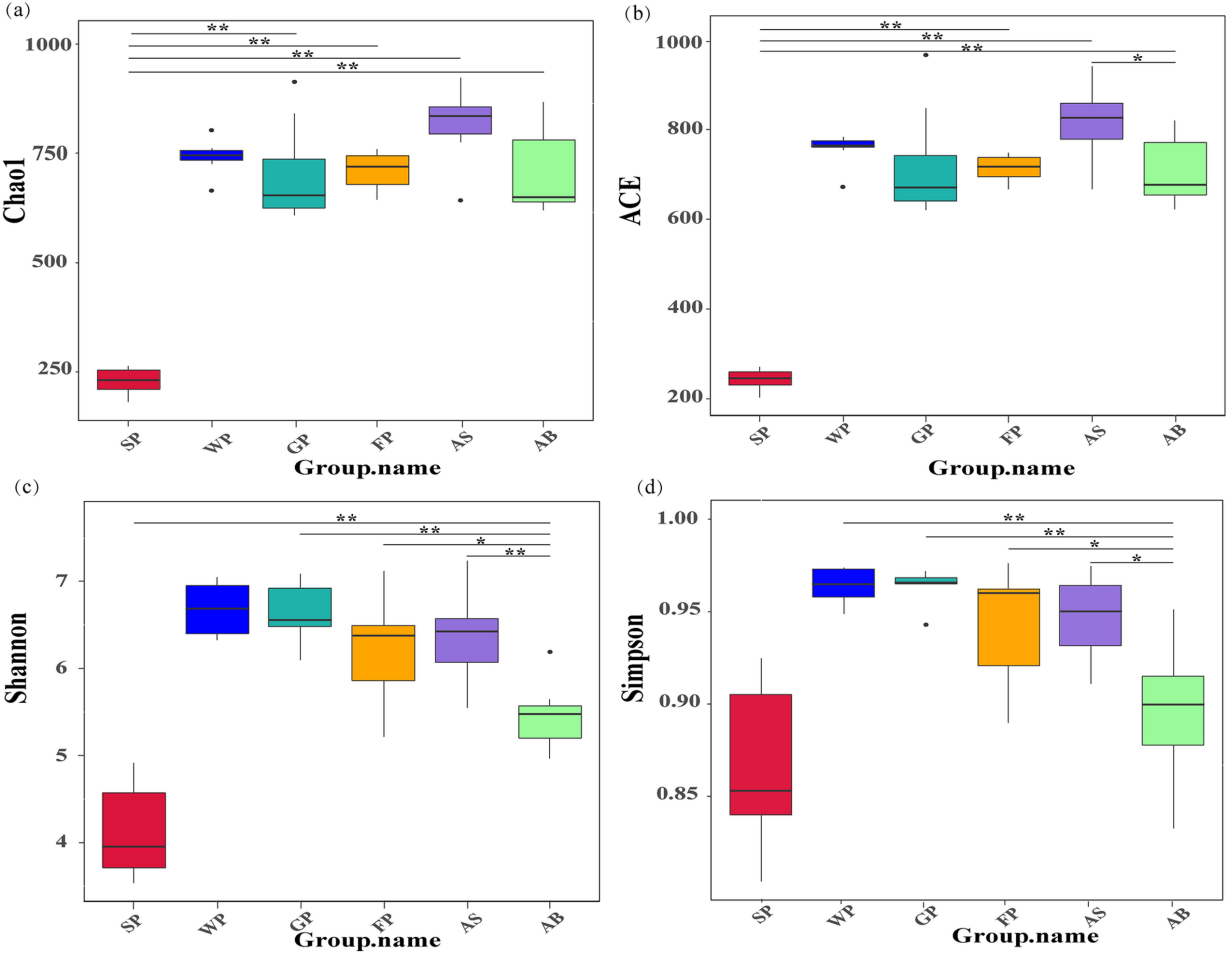
*α* diversity index of microbiota in Hezuo pigs at different ages **(a)** is the Chao1 index, which is used to estimate the total number of species contained in the community samples and can well reflect the presence of low-abundance species in the community. **(b)** is the ACE index (Abundance Coverage-based Estimator), which is used to estimate the number of OTUs in the community and can better reflect the overall situation of the community. **(c)** is Shannon’s diversity index: the higher the community diversity, the more uniform the species distribution, the greater the Shannon index; **(d)** is Simpson’s Index: by calculating the probability that two randomly sampled individuals belong to different species, the diversity and evenness of species distribution in the community are characterized. “*” indicates *p* < 0.05, and “* *” indicates *p* < 0.01.

Figure 1 displays the results of the α-diversity analysis of the fecal microbiota of Hezuo pigs across various ages. The ACE and Chao1 index plots indicated that the species richness of the intestinal microbiota of SP was significantly lower than that of the other groups (*p* < 0.01). Weaned piglets, growing pigs, and fattening pigs showed no significant differences in species richness and evenness (*p* > 0.05). These finding suggest that the intestinal microbiota Macrobiotic of newborn piglets was not fully colonized at birth. As the piglets mature and adapt to their environment and diet, the richness of their intestinal microbiota increases and stabilizes. Additionally, the analysis revealed that the intestinal microbiota of adult boars (AB) was significantly less diverse and homogeneous than that of adult sows (*p* < 0.05). This highlights potential gender-based differences in the microbial composition of the Microbiota in Hezuo pigs.

#### 3.2.4 PCoA analysis of intestinal microbiota in Hezuo pigs across different ages

**Figure 2:**
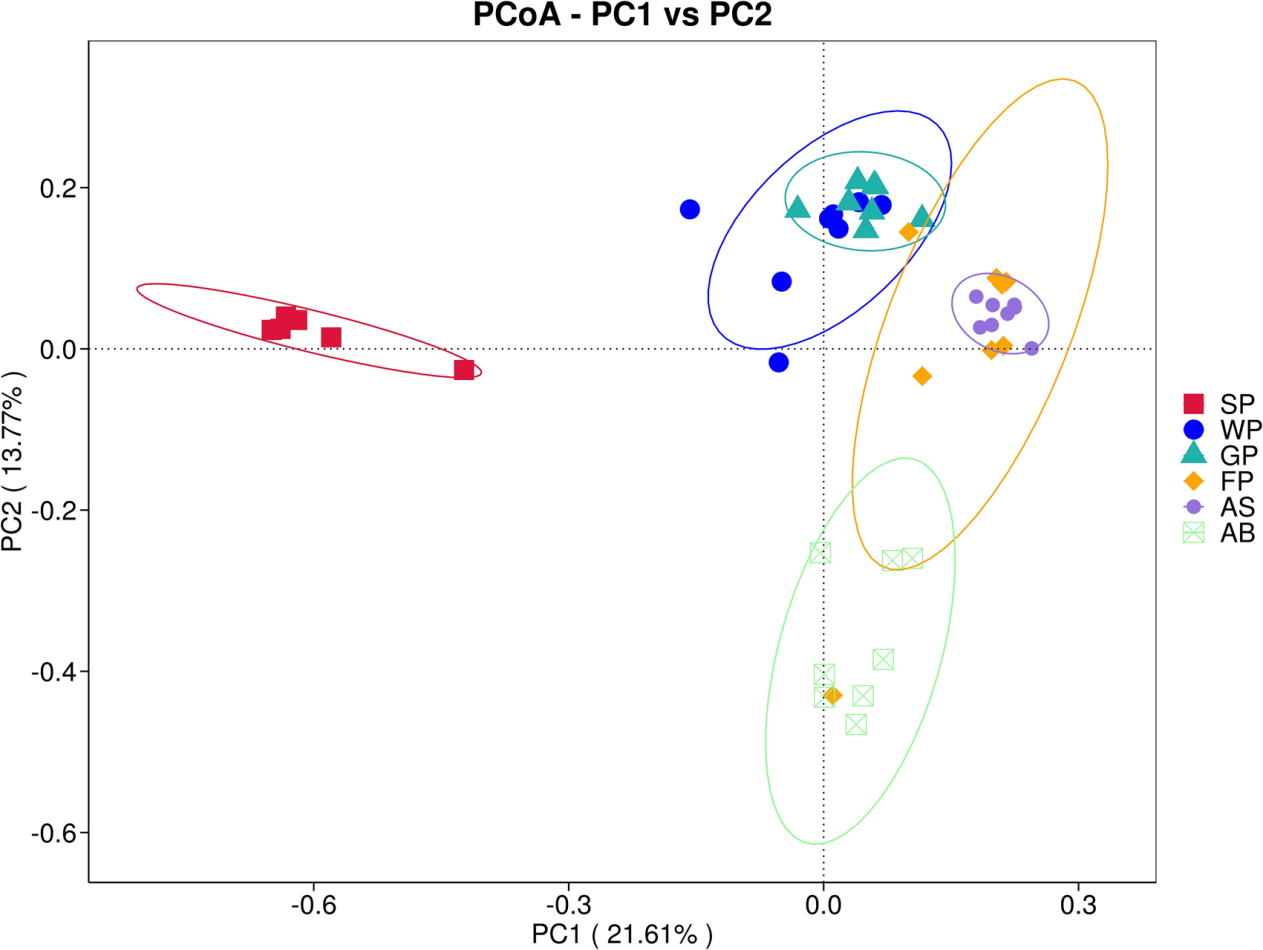
PCoA analysis of microbiota in Hezuo pigs across different ages based on Bray_Curtis distance. If the sample distance is closer, the species composition structure is more similar, so the samples with high community structure similarity tend to gather together, and the samples with great community difference will be far away.

The microbial compositions of suckling piglets and adult baors significantly differed from those of other developmental stages in Hezuo pig, as demonstrated by the principal component analysis (PCoA) based on Bray-Curtis distance at the OTU level. The contribution values of the two principal components, PC1 (21.61%) and PC2 (13.77%), are illustrated in Figure 2. The SP (suckling piglets)and AB (adult boars) groups were distinctly separated from the other groups, indicating substantial differences in their microbial profiles. In contrast, the WP (weaned piglets) group and GP (growing pigs) group were closely clustered, as were the FP (fattening pigs) and AS (adult sows) groups, suggesting minimal differences in gut microbial composition between these respective pairs. The WP (weaned piglets) group exhibited a more dispersed sample distribution, with greater distance between individual samples.

### 3.3 Analysis of intestinal microflora structure of Hezuo pigs at different ages

**Figure 3:**
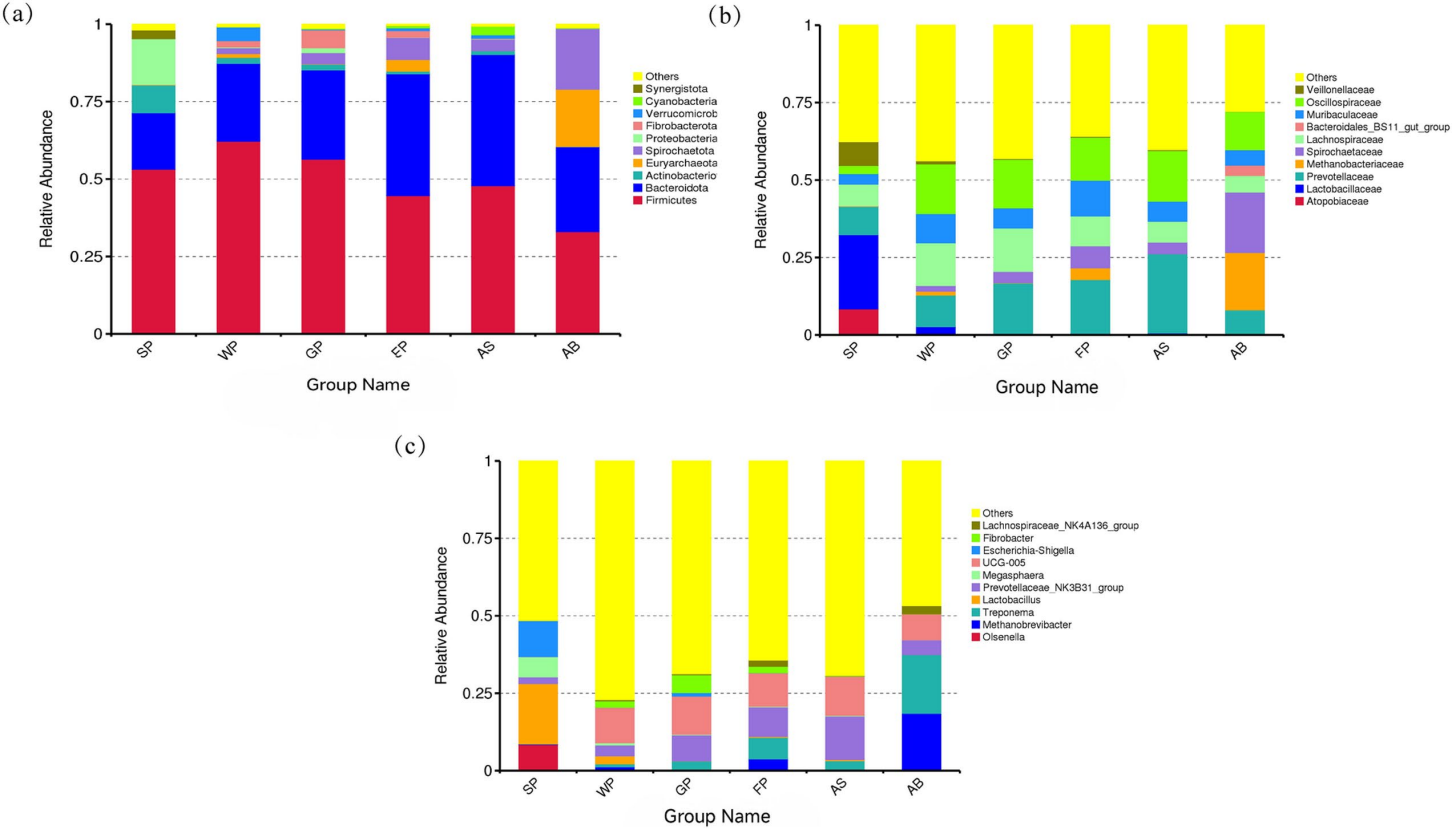
The relative abundance of top 10 microbiota in Hezuo pigs at different age stages at phyla **(a)**, family **(b)** and genus **(c)** levels.

Figure 3 illustrates the comparative abundance of the top 10 intestinal microbiota at the phylum, family, and genus levels in Hezuo pigs across different age groups. At the phylum level, Firmicutes and Bacteroidetes were the predominant intestinal microbiota, as shown in Figure 3a. These two phyla collectively accounted for over 60% of the intestinal microbiota in all six groups of Hezuo pigs. Spirochaetota was more prevalent in weaned piglets, growing and fattening pigs, adult sows, and adult boars. Suckling piglets exhibited a higher abundance of Proteobacteria and Actinobacteriota, while Euryarchaeota was the predominant phylum in adult boars.

At the family level, Hezuo pigs of varying ages shared several dominant bacterial families, including *Prevotellaceae*, *Oscillospiraceae*, *Lachnospiraceae*, and *S24-7 (Muribaculaceae)*, as depicted in Figure 3b. Unique to lactating piglets were the dominant families *Veillonellaceae*, *Lactobacillaceae*, and *Atopobiaceae*. In contrast, *Spirochaetaceae* and *Methanobacteriaceae* were characteristic of adult boars.

At the genus level, the intestinal microbiota of Hezuo pigs across different age groups displayed significant variation, as illustrated in Figure 3c. The dominant genus shared by all six groups was *Prevotellaceae_NK3B31_group*. Additionally, *UCG-005* from the Ruminococcaceae family was a dominant genus in the other five groups, excluding lactating piglets, with an average abundance exceeding 10%. In lactating piglets, the most prevalent genera were *Escherichia-Shigella*, *Lactobacillus*, *Megasphaera*, and *Olsenella*. In adult boars, the dominant genera *Treponema* and *Methanobrevibacter* exhibited colony abundances of over 18% (Figure 3c). These findings underscore the dynamic nature of microbial composition at differential taxa level, reflecting the influence of developmental stages and dietary transitions on the intestinal microbiota of Hezuo pigs.

### 3.4 Analysis of differences in intestinal microbiota composition of Hezuo pigs at different ages

#### 3.4.1 Intestinal microbiota markers of Hezuo pigs at different ages

**Figure 4:**
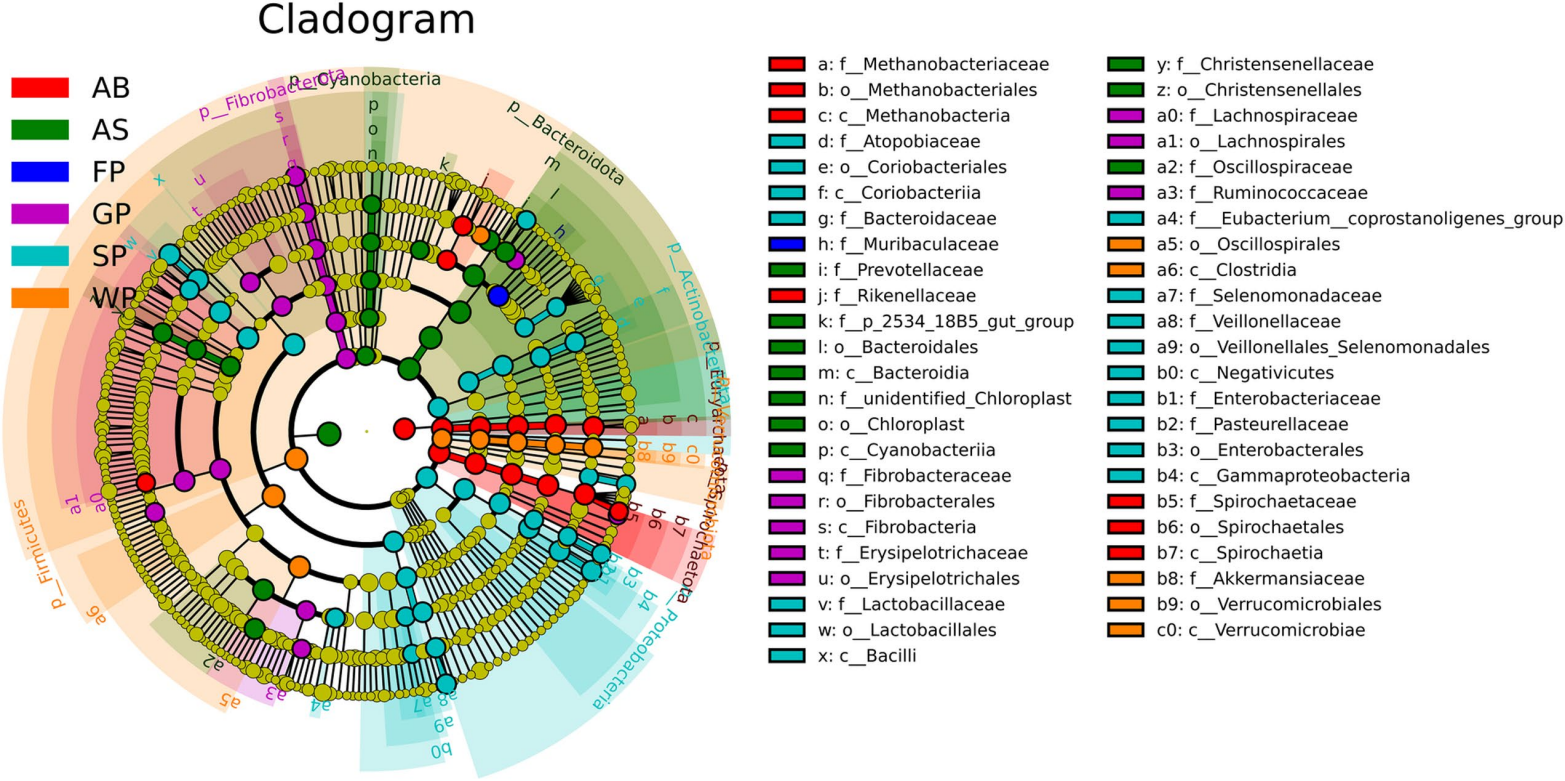
Evolutionary branch diagram of microbiota in Hezuo pigs at different ages. The circles radiating from the inside to the outside represent the classification level from the phylum to the genus (or species). The diameter of the small circle is proportional to the relative abundance.

The evolutionary branching diagram displayed 47 significant bacterial taxa and their effects, as determined by LDA scores (Figure 4; Supplementary material 2). These findings were derived from a detailed analysis of intestinal microbiota markers in Hezuo pigs at various ages using LEfse. The marker flora branches of Verrucomicrobiota, Verrucomicrobiae, Verrucomicrobiales, *Akkermansiaceae*, and *Akkermansia muciniphila* were identified during the weaning stage of Hezuo piglets. In lactating piglets, the intestinal microbiota markers included *Lactobacillus*, *Megasphaera*, and *Pasteurella*. For growing pigs, the primary markers were *Fibrobacter*, *Subdoligranulum*, and *Alloprevotella*. Fattening pigs were characterized by markers from the S24-7 family. In adult sows, the predominant markers were *UCG_005* from the Ruminococcaceae family and multiple instances of *Prevotellaceae_NK3B31_group*. Adult boars were primarily marked by *Methanobrevibacter*, *Treponema*, and *Lachnospiraceae_NK4A136_group*, among others.

#### 3.4.2 Differences in relative abundance of intestinal microbiota between adjacent age groups in Hezuo pigs

**Figure 5:**
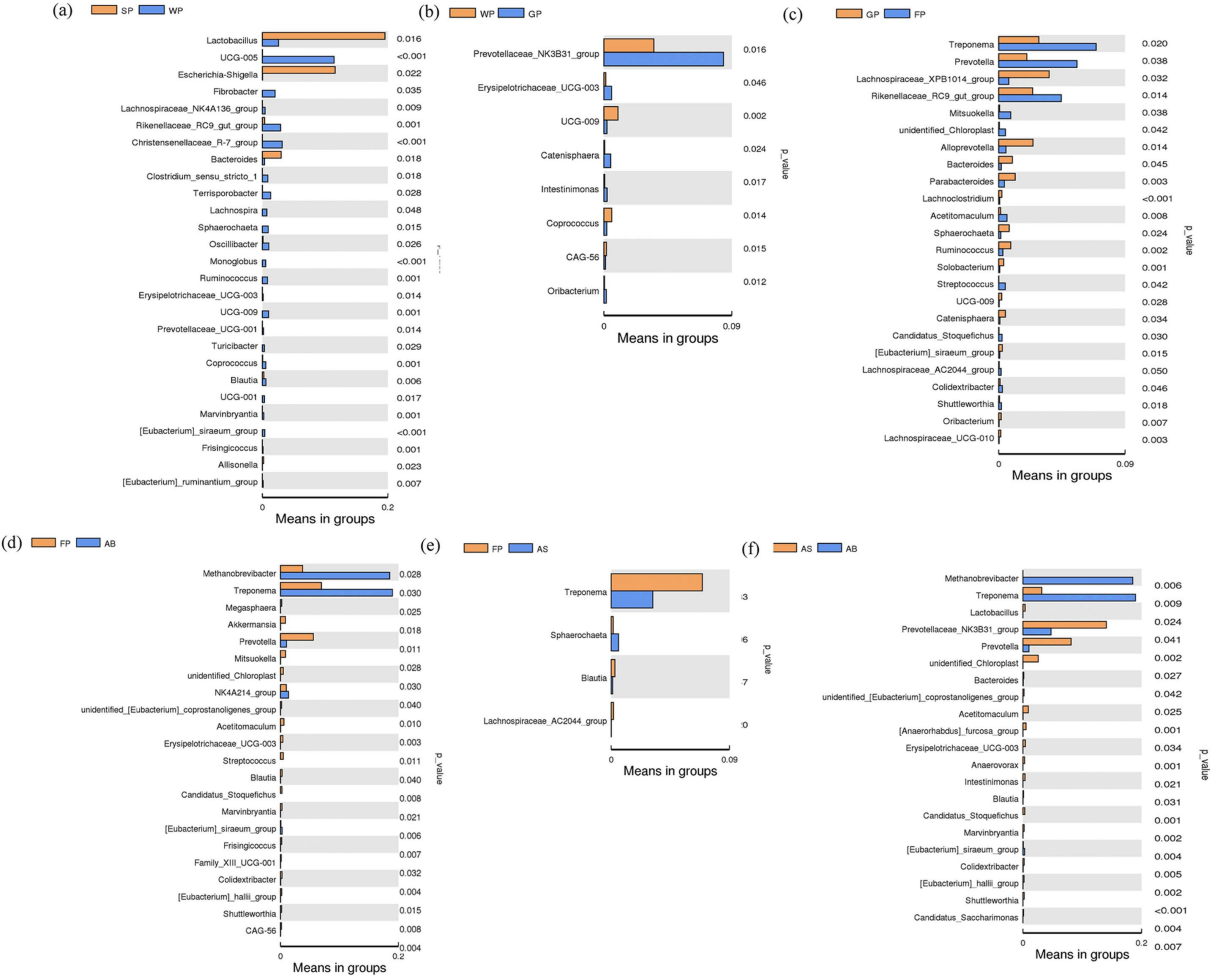
*T*-test difference analysis of microbial flora at genus level in adjacent age stages of Hezuo pig. **(a)** shows the difference in the relative abundance of microflora between suckling piglets and weaned piglets; **(b)** shows the difference in the relative abundance of microflora between weaned piglets and growing pigs; **(c)** shows the difference in the relative abundance of microorganisms between growing pigs and fattening pigs; **(d)** shows the difference in the relative abundance of microorganisms between fattening pigs and adult boars; **(e)** shows the difference in the relative abundance of microorganisms between fattening pigs and adult sows; and **(f)** shows the difference in the relative abundance of microorganisms between adult sows and adult boars.

A *T*-test was conducted to examine changes in the intestinal microbiota among Hezuo pigs of different ages, focusing on the predominant bacterial species in their intestinal tracts (Figure 5). The findings indicated that the relative abundance of *Lactobacillus*, *Escherichia-Shigella*, and *Bacteroidetes* in the intestinal tract of lactating piglets was significantly higher than that of weaned piglets (*p* < 0.05), while the relative abundance of *UCG-005* was markedly lower in lactating piglets compared to weaned piglets (*p* < 0.01, Figure 5a). The relative abundance of *Prevotellaceae_NK3B31_group* in growing pigs was considerably elevated compared to weaned piglets (*p* < 0.05). However, no significant differences were observed in this genus between lactating and weaned piglets, nor between growing and fattening pigs (Figures 5a–c). This may be related to the dietary and environmental changes that Hezuo pigs undergo during the transition from weaning to the growth phase.

In fattening pigs, the relative abundances of *Prevotella* spp., *Treponema* spp., and *rikenellaceae_RC9_gut_group* were significantly higher compared to growing pigs (*p* < 0.05, Figures 5b,c).

When comparing fattening pigs with adult boars and adult sows, substantial differences were observed in the relative abundance of 22 and 4 bacterial genera, respectively. Notably, the genera *Methanobrevibacter* and *Treponema* were substantially more abundant in the intestinal tract of adult boars than in fattening pigs (*p* < 0.05). Conversely, the relative abundance of *Treponema* in fattening pigs was significantly higher than in adult sows (*p* < 0.05). Additionally, the relative abundance of *Prevotella* was substantially higher in fattening pigs compared to adult sows (*p* < 0.01, Figures 5d,e).

The comparison of intestinal bacterial genera between adult boars and adult sows indicated that the levels of *Methanobrevibacter* and *Treponema* were significantly elevated in adult boars (*p* < 0.05), while the relative abundance of *Prevotella* and *Prevotellaceae_NK3B31_group* was significantly greater in adult sows (*p* < 0.05, Figure 5f).

### 3.5 Functional differences of intestinal microbiota in Hezuo pigs of adjacent age groups

**Figure 6:**
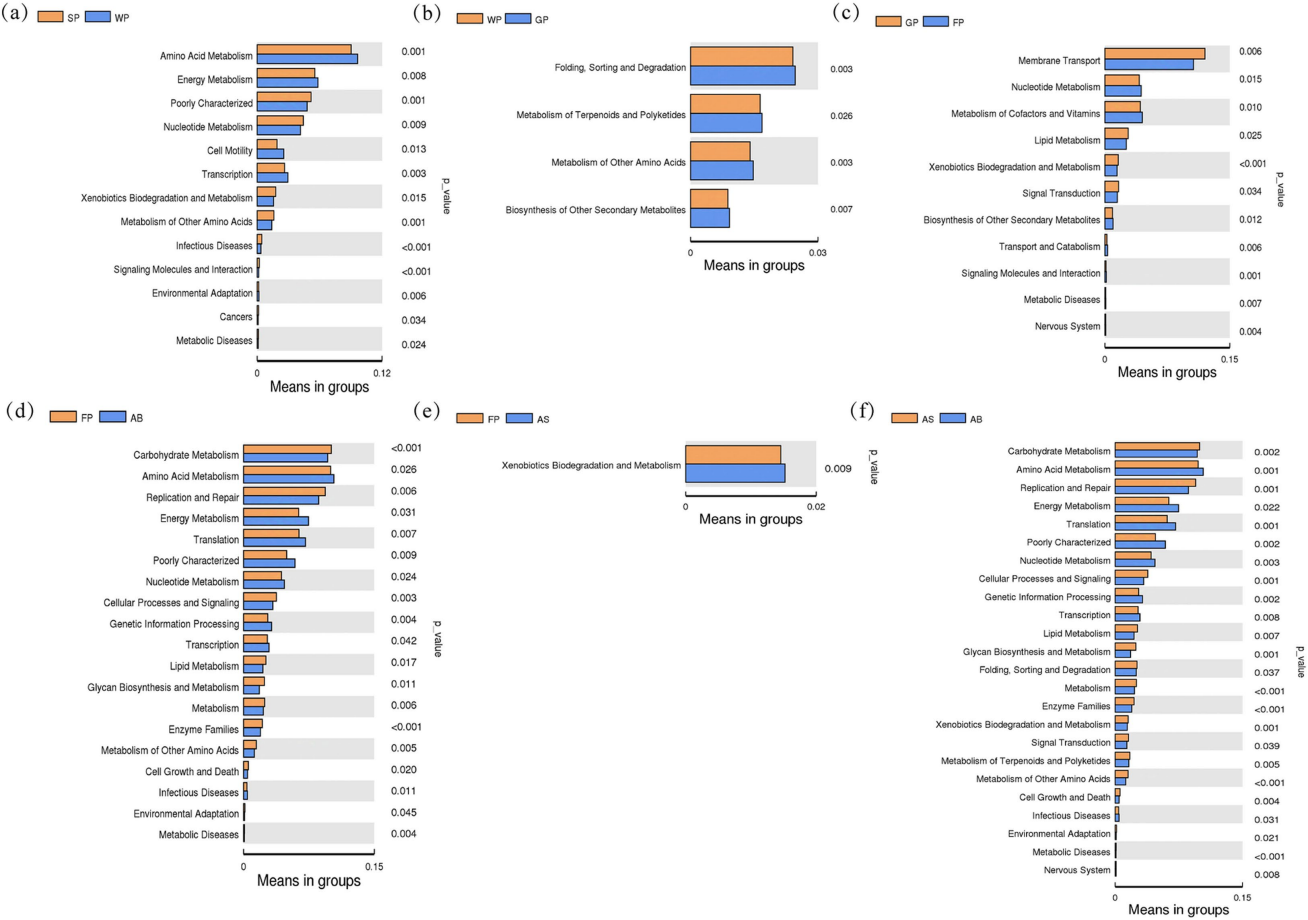
Difference analysis for the PICRUST function of intestinal microbiota in Hezuo pigs between adjacent age stages. **(a)** is the functional difference map between suckling piglets and weaned piglets; **(b)** is the functional difference map between weaned piglets and growing pigs; **(c)** is the functional difference map between growing pigs and fattening pigs; **(d)** shows the functional difference between fattening pigs and adult boars; **(e)** shows the functional difference between fattening pigs and adult sows; **(f)** shows the functional difference between adult sows and adult boars.

Functional genomic analysis of intestinal microbiota in cohabitating swine across adjacent developmental stages, conducted via PICRUSt software (Figure 6), revealed distinct metabolic pathway stratifications. Suckling piglets demonstrated pronounced enrichment in nucleotide metabolism, xenobiotic biodegradation, and secondary metabolite biosynthesis pathways compared to weaned counterparts (*p* < 0.01), whereas weaned piglets exhibited superior representation in amino acid and energy metabolism pathways (Figure 6a). Subsequent developmental transitions manifested dynamic alterations in differential pathways: the weaning-growth phase comparison showed reduced pathway divergence 4 pathways (including folding/sorting/degradation and terpenoid/polyketide metabolism), all exhibiting elevated genomic enrichment in growth-stage subjects (*p* < 0.01, Figure 6b). This trend reversed during growth-finishing transitions, with 11 divergent pathways emerging. Finishing-stage specimens displayed diminished transmembrane transport and signal transduction pathway representation (*p* < 0.05), yet exhibited enhanced nucleotide metabolism, cofactor biosynthesis, and secondary metabolite pathway enrichment (*p* < 0.05, Figure 6c). Notably, secondary metabolite pathway enrichment demonstrated a progressive ascending trajectory from weaning through finishing phases (Figures 6a–c).

In the comparison between fattening pigs and adult sows, only the xenobiotic degradation and metabolism pathways was significantly more gene-enriched than in adult sows (*p* < 0.01, Figure 6e). Also, this pathway was significantly less gene-enriched in the fattening stage than in the growing stage (*p* < 0.01, Figure 6c). Most striking genomic disparities emerged between fatting pigs and adult boars, with the latter showing marked depletion in carbohydrate/lipid metabolism, cellular processes/signaling, and replication/repair pathways (*p* < 0.01, Figure 6d), whereas that of amino acid/energy/nucleotide metabolism and genetic information processing pathway was elevated enrichment (*p* < 0.05, Figure 6d). Notably, the genetic information processing pathways was significantly enriched only in adult boars, suggests a potential association Hezuo pig’s reproductive physiology.

Gender-specific profiling unveiled profound metabolic dimorphism: adult boars exhibited superior enrichment in amino acid/energy/nucleotide metabolism and genetic information processing pathways than adult sows (*p* < 0.01), while demonstrating reciprocal depression in carbohydrate/lipid metabolism and replication/repair pathways than in adult sows (*p* < 0.01, Figure 6f).

## 4 Discussion

The present study revealed that the intestinal microbiota of Hezuo piglets was primarily composed of Firmicutes and Bacteroidetes, and the relative abundance of Bacteroidetes increasing with age. Bacteroidetes exhibit robust metabolic capabilities in the gastrointestinal tract of animals, particularly in carbohydrate metabolism, converting complex carbohydrates into assimilable monosaccharides and producing short-chain fatty acids (SCFAs) (Lapébie et al., 2019; Vera-Ponce de León et al., 2020). During lactation, piglets rely on protein-rich milk, correlating with higher intestinal Proteobacteria abundance (associated with protein fermentation) (Vijande et al., 2024). As piglets transition to solid feed, carbohydrate intake rises, driving Bacteroidetes proliferation, consistent with their growth trajectory. In the PCoA diagram, the suckling piglets group was farther away from other groups, which also reflected that the piglets experienced significant environmental and dietary changes during the transition from breastfeeding to roughage feeding.

Notably, the relative abundance of Firmicutes in Hezuo pigs peaked at only 62% across five developmental stages of lactation, weaning, growth, fattening, and maturity, significantly lower than in “Duroc×Landrace×Yorkshire” (DLY) pig (79% at fattening stage) (Huang et al., 2020) and Landrace pigs in the (74% at adulthood stage) (Xiao et al., 2018). This reduced Firmicutes abundance aligns with their lower fattening efficiency compared to foreign pig breeds, as Firmicutes enrichment is linked to enhanced fat deposition (Takeuchi et al., 2023), Thus, the diminished Firmicutes:Bacteroidetes ratio in Hezuo pigs may explain their reduced adipogenic potential, highlighting microbiota-driven metabolic adaptations in breed-specific growth patterns.

The predominant bacterial genus in the gastrointestinal tracts of Hezuo pigs varied significantly across age groups, yet the *Prevotella_NK3B31_group* consistently dominated at the genus level. As a key fiber-degrading genus, *Prevotella* facilitates dietary fiber breakdown and enhances energy harvest efficiency from plant polysaccharides (Christensen et al., 2019). Its abundance is strongly associated with improved intestinal health, including reduced inflammation and lower risks of non-infectious colonic disorders (Christensen et al., 2019). Notably, *Prevotella*-enriched microbiota produce 2 to 3 times more propionic acid (a critical short-chain fatty acid) from arabinoxylan and oligofructose compared to *Bacteroidetes*-dominant communities (Tett et al., 2021). The sustained high abundance of *Prevotella* across all developmental stages in Hezuo pigs likely optimizes their adaptation to high-fiber diets by enhancing nutrient extraction and metabolic efficiency.

The relative abundance of Ruminococcaceae genus UCG-005 was significantly higher in weaned Hezuo pigs compared to suckling piglets, with this cellulose-degrading bacterium persisting through growth stages in plateau-raised Hezuo pigs. Studies have indicated that UCG-005 has the ability to secrete cellulose-degrading enzymes, which is crucial for starch and cellulose digestion in animals (Sun et al., 2019). Its prevalence aligns with Hezuo pigs’ high-fiber pasture diet that an adaptation supported by evidence showing increased Ruminococcaceae abundance in high-altitude animals (Du et al., 2022). Contrastingly, gut microbiota composition differed between breeds. Huang et al. Identified that *Lactobacillus* spp. and *Streptococcus* spp. are the predominant genera in the gut of DLY pigs during fattening stage (Huang et al., 2020). *Lactobacillus* and *Clostridium* species are the most prevalent genera in the gut of adulthood Landrace pigs. *Lactobacillus* have been shown to enhance nutrient absorption and daily weight gain while reducing jejunal *Escherichia coli* levels in Landrace pigs (Xiao et al., 2018). In this study, Hezuo pigs showed notably lower *Lactobacillus* levels at fattening and adulthood stages compared to DLY pigs and Landrace pigs during equivalent stages (Konstantinov et al., 2008; Yang et al., 2020). Given Lactobacillus’ documented role in enhancing nutrient absorption and daily weight gain, this deficiency may explain Hezuo pigs’ slower growth rates during later development phases.

The intestinal microbiota of lactating Hezuo piglets is dominated by *Escherichia-Shigella*, a genus containing primary pathogens of bacillary dysentery (Matanza and Clements, 2023). This colonization may underpin the heightened susceptibility of neonatal piglets to diarrheal diseases. In contrast, weaned piglets exhibit a marked enrichment of *Akkermansia muciniphila*, a mucinophilic bacterium recognized as a microbial biomarker during this transitional phase. *Akkermansia muciniphila* beyond its role as an indicator, *Ackermannia muciniphila* demonstrates therapeutic potential in modulating metabolic dysregulation, with evidence linking its abundance to reduced risks of obesity, diabetes, and cardiovascular disorders (Xu et al., 2020). Critically, its proliferation during weaning coincides with immune system maturation, suggesting a synergistic role in both metabolic homeostasis and immunological resilience.

Notably, adult Hezuo boars display a distinct gut microbiota profile compared to sows, characterized by significant enrichment of *Methanobrevibacter* and *Treponema*. *Methanobrevibacter*, a hydrogenotrophic methanogenic bacterium, drives cellulose degradation in feedstuffs (Zhang et al., 2021), while *Treponema* specializes in pectin breakdown within fibrous diets (Baniel et al., 2021). This functional synergy not only enhances microbial protein synthesis but also optimizes energy harvest efficiency. The metabolic byproducts of these bacteria, such as SCFAs and methane, may serve dual purposes: sustaining body mass through caloric extraction and supporting reproductive fitness via protein accretion. Such adaptations likely reflect evolutionary strategies to meet the elevated energetic demands of growth, somatic maintenance, and spermatogenesis in Hezuo boars. Also, significant enrichment of the genetic information processing pathway in adult boars was found in the functional prediction of gut flora.

Age-dependent alterations in fiber-digesting enzyme activities in Hezuo pigs mirror dynamic changes in their gut microbiota composition. Cellulose, a complex polymer of D-glucose molecules, linked by interconnected *β*-1,4-glycosidic bonds (Dutta and Bhat, 2022), requires specialized enzymatic machinery for degradation. As a key cellulose-degrading enzymes, β-glucosidases catalyze the hydrolysis of β-D-glucoside bonds, facilitate the hydrolysis of β-D-glucoside residues, initiating cellulose depolymerization (Godse et al., 2021). Notably, intestinal β-glucosidase activity in Hezuo pigs demonstrates an age-associated increase, peaking in adult boars where enzymatic levels surpass those in sows by 1.5- to 2-fold. This sexual dimorphism aligns with the distinct microbial ecology of adult boars, which harbor a richer diversity of fibrolytic bacteria (e.g., *Ruminococcus* and *Fibrobacter*) alongside methanogens like *Methanobrevibacter*.

## 5 Conclusion

This study delineates age-dependent shifts in the gut microbiota of Hezuo pigs, revealing Firmicutes and Bacteroidetes as the dominant phyla across all life stages, with Prevotella_NK3B31_group consistently emerging as a core genus. Notably, Ruminococcaceae_UCG-005 dominates microbial communities during weaning, growth, fattening and adulthood. Functional profiling demonstrates progressive enrichment of secondary metabolite biosynthesis genes throughout pre-adult developmental phases (weaning to fattening), while genetic information processing pathway, particularly those linked to DNA repair and replication, predominate in adulthood Hezuo pigs. These findings illuminate the characteristics underlying the enrichment of specific microbial flora in Hezuo pigs of all ages, providing a mechanistic basis for their exceptional adaptability to roughage. This will help reduce breeding costs, improve resource utilisation efficiency and environmental benefits, and make it competitive in cost-sensitive markets and ecological agriculture.

## Data Availability

The original contributions presented in the study are publicly available. This data can be found here: https://www.ncbi.nlm.nih.gov/, accession number: PRJNA1199767.
