# Supplementary material for: Cellulase activity and age-based variation of intestinal microbiota in Hezuo pigs
Source: Front Microbiol. 2025 May 9;16:1599847. doi: 10.3389/fmicb.2025.1599847 (PMC12101066; doi:10.3389/fmicb.2025.1599847)
Supplement: Supplementary file 1 [file Supplementary_file_1.docx]

| Analyze content | Software | version | parameters (Functions, thresholds, URLs, or computer programming languages used by the software) |
| --- | --- | --- | --- |
| Data splitting | python | 3.6.13 |  |
| Filtering | cutadapt | 3.3 | --minimum-length |
| Splicing | flash | 1.2.11 | --min-overlap |
| Quality control | fastp | 0.23.1 | The default parameter of -n is used to screen the proportion of N bases in fastp software. When the number of N bases in the default read is greater than 5, it is eliminated. In addition, the setting threshold of -q is 19; that is, the quality value is greater than or equal to Q19 to be qualified. The threshold control of low-quality ratio adopts the parameter of -u, and the threshold is set to 15, which allows the proportion of unqualified bases below 15 % to exist. |
| De-chimera | vsearch | 2.16.0 | The Tags obtained by quality control were compared with the database (16S/18S: Silva database, ITS : Unitedatabase ) to detect the chimera sequence and remove it (http://www.drive5.com/usearch/manual/chimera_formation.html). |
| Visualization of dilution curve | R | 4.0.3 | plyr、reshape2、ggplot2 |
| Alpha diversity analysis | Qiime | 1.9.1 | The indices for calculating community richness were as follows:  Chao-theChao1estimator (http://www.mothur.org/wiki/Chao);  ACE-the ACE estimator (http://www.mothur.org/wiki/Ace).  The indices for calculating community diversity are:  Shannon-the Shannon index (http://www.mothur.org/wiki/Shannon); Simpson-the Simpson index (http://www.mothur.org/wiki/Simpson).  The indexes for calculating the sequencing depth are:  Coverage-the Good 's coverage (http://www.mothur.org/wiki/Coverage). |
| PCoA analysis | R | 4.0.3 | ggplot2、extrafont、grid、ade4 |
| top10 histogram | perl | 5.26.2 | SVG |
| lefse | lefse | 1.1.01 | -l |
| ttest | R | 4.0.3 |  |
| picrust | picrust | 1.1.4 |  |
