# Supplementary material for: Cellulase activity and age-based variation of intestinal microbiota in Hezuo pigs
Source: Front Microbiol. 2025 May 9;16:1599847. doi: 10.3389/fmicb.2025.1599847 (PMC12101066; doi:10.3389/fmicb.2025.1599847)
Supplement: Supplementary file 2 [file Supplementary_file_2.docx]

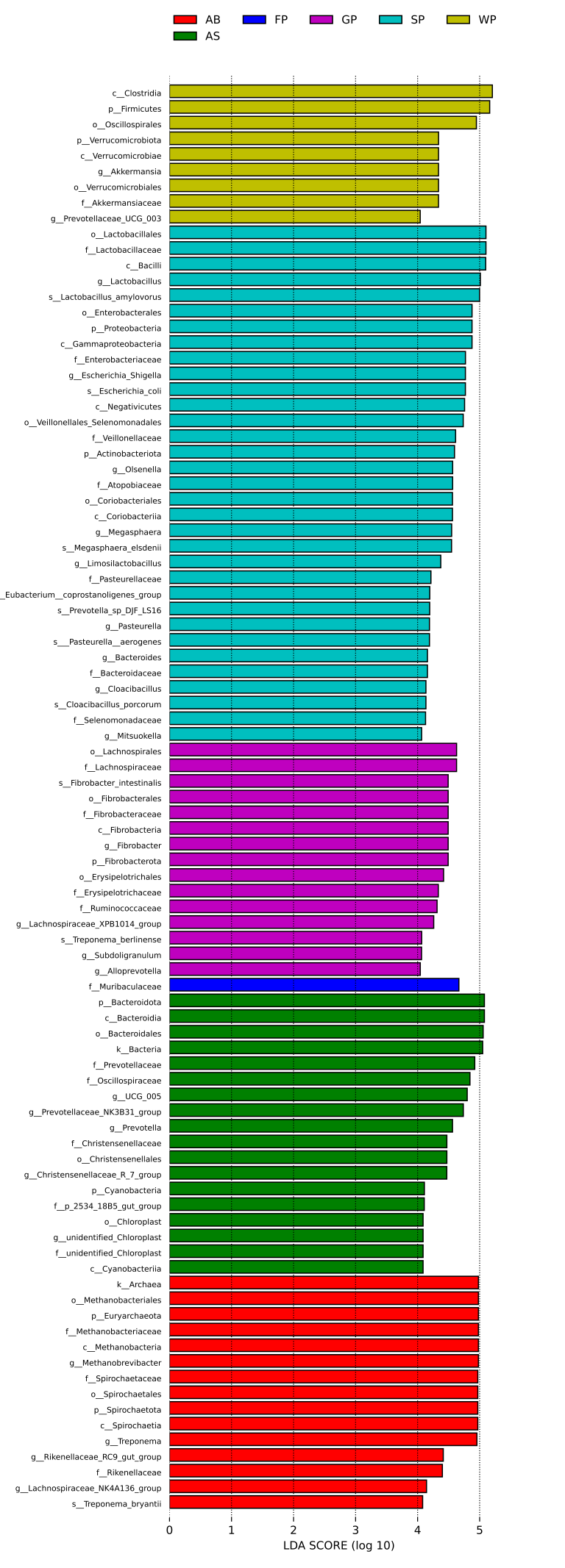


Distribution histogram of LDA values of intestinal microbiota in Hezuo pigs at different ages Note. The Figureure shows the species with significant differences in abundance (LDA score>4.0) in each age group. The length of histogram bars represents LDA score.
